# Systematic MicroRNA Analysis Identifies ATP6V0C as an Essential Host Factor for Human Cytomegalovirus Replication

**DOI:** 10.1371/journal.ppat.1003820

**Published:** 2013-12-26

**Authors:** Jon Pavelin, Natalie Reynolds, Stephen Chiweshe, Guanming Wu, Rebecca Tiribassi, Finn Grey

**Affiliations:** 1 Division of Infection and Immunity, The Roslin Institute, University of Edinburgh, Easter Bush, Midlothian, United Kingdom; 2 OCTRI, Health Sciences University, Portland, Oregon, United States of America; 3 Vaccine and Gene Therapy Institute, Health Sciences University, Portland, Oregon, United States of America; University of Alabama at Birmingham, United States of America

## Abstract

Recent advances in microRNA target identification have greatly increased the number of putative targets of viral microRNAs. However, it is still unclear whether all targets identified are biologically relevant. Here, we use a combined approach of RISC immunoprecipitation and focused siRNA screening to identify targets of HCMV encoded human cytomegalovirus that play an important role in the biology of the virus. Using both a laboratory and clinical strain of human cytomegalovirus, we identify over 200 putative targets of human cytomegalovirus microRNAs following infection of fibroblast cells. By comparing RISC-IP profiles of miRNA knockout viruses, we have resolved specific interactions between human cytomegalovirus miRNAs and the top candidate target transcripts and validated regulation by western blot analysis and luciferase assay. Crucially we demonstrate that miRNA target genes play important roles in the biology of human cytomegalovirus as siRNA knockdown results in marked effects on virus replication. The most striking phenotype followed knockdown of the top target ATP6V0C, which is required for endosomal acidification. siRNA knockdown of ATP6V0C resulted in almost complete loss of infectious virus production, suggesting that an HCMV microRNA targets a crucial cellular factor required for virus replication. This study greatly increases the number of identified targets of human cytomegalovirus microRNAs and demonstrates the effective use of combined miRNA target identification and focused siRNA screening for identifying novel host virus interactions.

## Author Summary

Human cytomegalovirus is a prevalent pathogen. Like other herpesviruses, human cytomegalovirus expresses small regulatory RNAs called microRNAs. The focus of this study was to understand the role of these RNAs in the context of viral infection and to use this information to identify novel host factors involved in human cytomegalovirus biology. We used a biochemical approach that allowed us to systematically identify cellular genes targeted by virus microRNAs. Because the virus targets these genes, it is reasonable to propose that these genes play an important role during infection. We confirmed this hypothesis using a second screen in which we knocked down expression of a number of the identified targets of the virus microRNAs. Knock down of one of the targets, a cellular factor called ATP6V0C, resulted in an almost complete block in production of infectious virus. These data suggest that endosomal acidification is crucial to HCMV replication, and the virus targets this process by microRNA regulation.

## Introduction

Human cytomegalovirus (HCMV) is a highly prevalent infectious disease, infecting greater than 30% of the population. Although normally asymptomatic in healthy individuals, HCMV infection is a significant cause of morbidity and mortality in immunocompromised populations, individuals with heart disease and recipients of solid organ and bone marrow transplants [1]–[8]. HCMV is also the leading cause of infectious congenital birth defects resulting from spread of the virus to the unborn fetus. Reactivation of virus from a latent infection, rather than primary infection, is often responsible for HCMV associated pathologies [9]–[13].

The capacity of HCMV to strictly regulate the expression of its own genes and to manipulate host gene expression is crucial to the virus's ability to replicate and its success in maintaining a persistent infection [14]. Studies in our lab and others have demonstrated that herpesviruses have evolved to encode microRNA (miRNA) genes, enabling regulation of the virus's gene expression profile as well as altering the host environment by targeting cellular transcripts. Recent reports have demonstrated roles for viral miRNAs in suppressing apoptosis, immune evasion and regulation of viral replication through targeting of both cellular and viral gene expression [15].

HCMV encodes at least 14 pre-miRNAs corresponding to a total of 27 mature miRNA species [16]–[20]. Clear functions have not been shown for the majority of HCMV miRNAs. However, these regulatory RNAs have been shown to target genes involved in viral latency, immune evasion, and cell cycle control [21]. We previously demonstrated that the HCMV miRNA, UL112-1, restricted viral acute replication through targeting of the major immediate early gene IE72, suggesting this miRNA may play a role in establishing and maintaining viral latency [22]. Others have since shown that targeting of immediate early genes by viral miRNAs may be a fundamental mechanism involved in herpesvirus latency regulation [23]–[26]. UL112-1 has also been shown to target the major histocompatibility complex class I-related chain B (MICB) resulting in reduced killing by NK cells [27].

Despite these advances, identification of miRNA targets remains challenging. Until we have a greater understanding of the rules governing miRNA target interaction, bioinformatic strategies alone continue to produce unreliable results, especially for viral miRNAs, which in most cases do not display significant evolutionary conservation.

Biochemical approaches have provided an alternative means for the identification of miRNA targets. One such approach, RISC immunoprecipitation (RISC-IP) has proved effective in identifying both cellular and viral targets [28]. Recently, we used a RISC-IP approach to identify multiple cellular targets of US25-1, an HCMV miRNA expressed at high levels during acute infection [29]. Here we use a combined approach of RISC-IP profiling in infected cells combined with focused siRNA screening to identify host targets of HCMV miRNAs that have significant effects on virus replication. Our results, using a laboratory strain as well as a clinical strain of virus greatly increases the number of identified and validated HCMV miRNA targets. Furthermore, the results show that the V-ATPase complex, involved in acidification of endosomal compartments, is essential for HCMV virus replication and is targeted by the HCMV miRNA US25-1.

## Results

**Figure 1:**
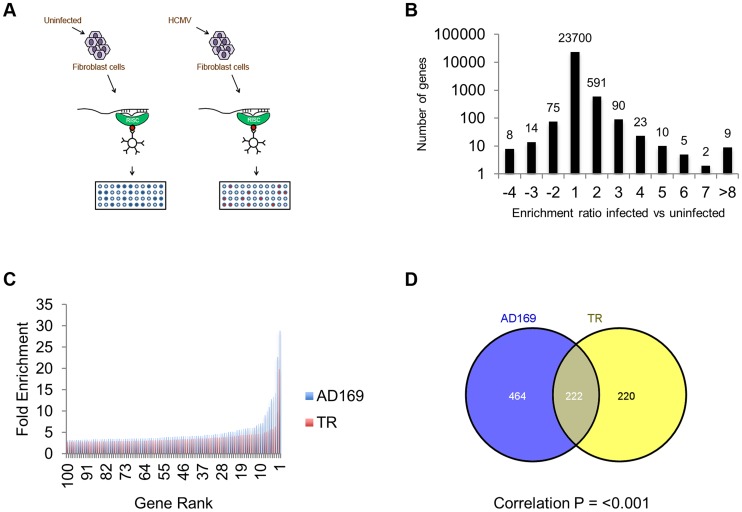
Systematic analysis of RISC-IP from HCMV infected fibroblast cells. (A) Schematic representation of RISC-IP procedure in HCMV infected and uninfected fibroblast cells. (B) Enrichment profile of all genes from AD169 infected cells. Genes were binned according to the enrichment ratio of infected vs uninfected. For example genes with an enrichment ratio from supplemental table 1 of between 0.5 and <2.0 were binned to 1 whereas genes with an enrichment profile of <0.5 but >0.33 were binned to −2. Total number of genes are shown above each bar. Values are skewed towards positive enrichment indicating effective enrichment of HCMV miRNA targets (C). Enrichment profile of the top 100 genes from cells infected with AD169 or TR. (D) Overlap of genes enriched greater than two fold between AD169 infected cells and TR infected cells. Correlation between the enriched profiles was highly significant as determined by Chi Squared test.

RISC-IP techniques have recently been used to identify targets of viral miRNAs [29], [30]. The approach relies on the stable interaction of the miRNA associated RISC protein complex with the targeted transcript. Following lysis of cells the RISC complexes are immunoprecipitated using direct antibodies that recognize Argonaute 2. RNA is then isolated, labeled and analysed by microarray to identify transcripts which are significantly enriched due to miRNA targeting. In a previous study we used this technique to identify targets of a single miRNA, US25-1, in the context of HEK293 cells [29]. Here we used the same basic approach to identify targets in primary human fibroblast cells infected with either the laboratory adapted AD169 strain of HCMV or the clinical strain TR. In both cases cells were infected at a high multiplicity of infection (MOI) of three and cells harvested three days post infection. Following lysis and immunoprecipitation, RNA was isolated by trizol extraction and analysed by microarray using the Illumina HumanRef-8 platform which contains probes for approximately 24,000 well annotated genes (Figure 1A). In addition to uninfected cells, RNA was analysed from infected cells immunoprecipitated with pre-immune serum instead of anti-Argonaute 2 antibody. To determine the level of enrichment, lysate was sampled before immunoprecipitation to establish total levels of transcript expression. Enrichment was then calculated as the transcript level of the IP sample divided by the total RNA sample. To determine transcripts specifically targeted by HCMV miRNAs, the level of enrichment from infected samples was divided by the level of enrichment in uninfected samples. However, infection with HCMV results in significant perturbation of total levels of many cellular transcripts through mechanisms unrelated to miRNA expression. False positive enrichment attributed to viral miRNA targeting can therefore occur due to down regulation of total RNA levels in the infected sample, where IP background levels remain relatively unchanged. To overcome this, a correction calculation was introduced using the results from the control IP using the pre-immune serum pull down. As enrichment values in this sample would only occur through changes in total levels due to AD169 infection, rather than any effective enrichment through specific immunoprecipitation, false enrichment could be effectively subtracted from the data sets generated with anti-Argonaute 2 pull downs. Example calculations are shown in supplemental figure S1.

The results indicate that greater than 96% of transcripts showed little or no enrichment in infected cells compared to uninfected cells, as would be expected if virus miRNAs are targeting a specific subset of transcripts (Figure 1B). In cells infected with AD169, 686 transcripts were enriched two fold or more with enrichment levels as high as 28.9 fold for the top target ATP6V0C. Enrichment levels were slightly lower for TR infected cells with 442 genes enriched 2 fold or higher with the highest level of enrichment for COMMD10 at 19.8 fold (Figure 1C). The lower enrichment in TR infected cells was expected as the clinical strain replicates less efficiently than AD169 in primary fibroblast cells, resulting in lower levels of miRNA expression (data not shown). Given that HCMV miRNAs are completely conserved between TR and AD169, with the exception of miR-148D-1, which is deleted from AD169 due to genome rearrangements, a similar suite of enriched genes would be expected from each pull down experiment. Indeed, 222 of the 442 genes enriched by two fold or more in cells infected with TR, were also enriched in the AD169 sample. This is a highly significant level of correlation (P = 3.2×10^−233^ as determined by hypergeometric distribution analysis) (Figure 1D) that validates the biological reproducibility of the system. Of the top 30 most highly enriched transcripts from AD169 infected cells, 26 were also enriched at least two fold in pull downs from TR infected cells, giving a high level of confidence that these genes are specifically enriched due to HCMV miRNA targeting. Table 1 lists the top 30 most highly enriched genes from cells infected with AD169 with corresponding enrichment values for TR. The complete data sets are shown in supplemental tables S1 and S2.

**Table 1:** Summary table of the top 30 most enriched genes following infection with AD169.

Gene	AD169	TR	DEFINITION
**ATP6V0C**	28.9	9.8	ATPase, H+ transporting, lysosomal
**GRN**	22.8	2.4	granulin
**COMMD10**	14.3	19.9	COMM domain containing 10
**CTRB2**	13.3	6.4	chymotrypsinogen B2
**SGSH**	12.8	3.4	N-sulfoglucosamine sulfohydrolase
**LGALS3**	10.9	3.6	galactoside-binding, soluble, 3 (galectin 3)
**PIGH**	9.9	3.7	phosphatidylinositol glycan anchor biosynthesis
**BSG**	9.0	1.7	basigin
**LEPRE1**	7.2	2.4	leucine proline-enriched proteoglycan
**LIN28B**	7.1	2.9	lin-28 homolog B
**SRPRB**	7.0	3.8	signal recognition particle receptor
**PDIA5**	6.6	4.2	protein disulfide isomerase family A
**CCNE1**	6.1	5.7	cyclin E1
**FLJ39061**	6.1	3.2	FLJ39061
**NUCB1**	6.0	0.9	nucleobindin 1
**PRSS8**	6.0	4.6	protease, serine, 8
**BCKDHA**	5.9	1.8	branched chain keto acid dehydrogenase E1
**C1ORF35**	5.9	4.7	chromosome 1 open reading frame 35
**TMEM74**	5.7	2.4	transmembrane protein 74
**NUCB2**	5.6	3.1	nucleobindin 2
**DSC2**	5.5	2.0	desmocollin 2
**FALZ**	5.2	2.1	fetal Alzheimer antigen
**CCNE2**	5.2	2.6	cyclin E2
**STX10**	5.1	3.5	syntaxin 10
**COL1A1**	5.0	3.4	collagen, type I
**KRTAP10-7**	4.9	4.1	keratin associated protein 10-7
**FAM38A**	4.9	2.3	family with sequence similarity 38
**ACP2**	4.7	1.8	acid phosphatase 2, lysosomal
**ATP1A3**	4.7	4.2	ATPase, Na+/K+ transporting
**PROK2**	4.7	4.4	prokineticin 2

Corresponding enrichment levels are shown for TR infected cells.

**Figure 2:**
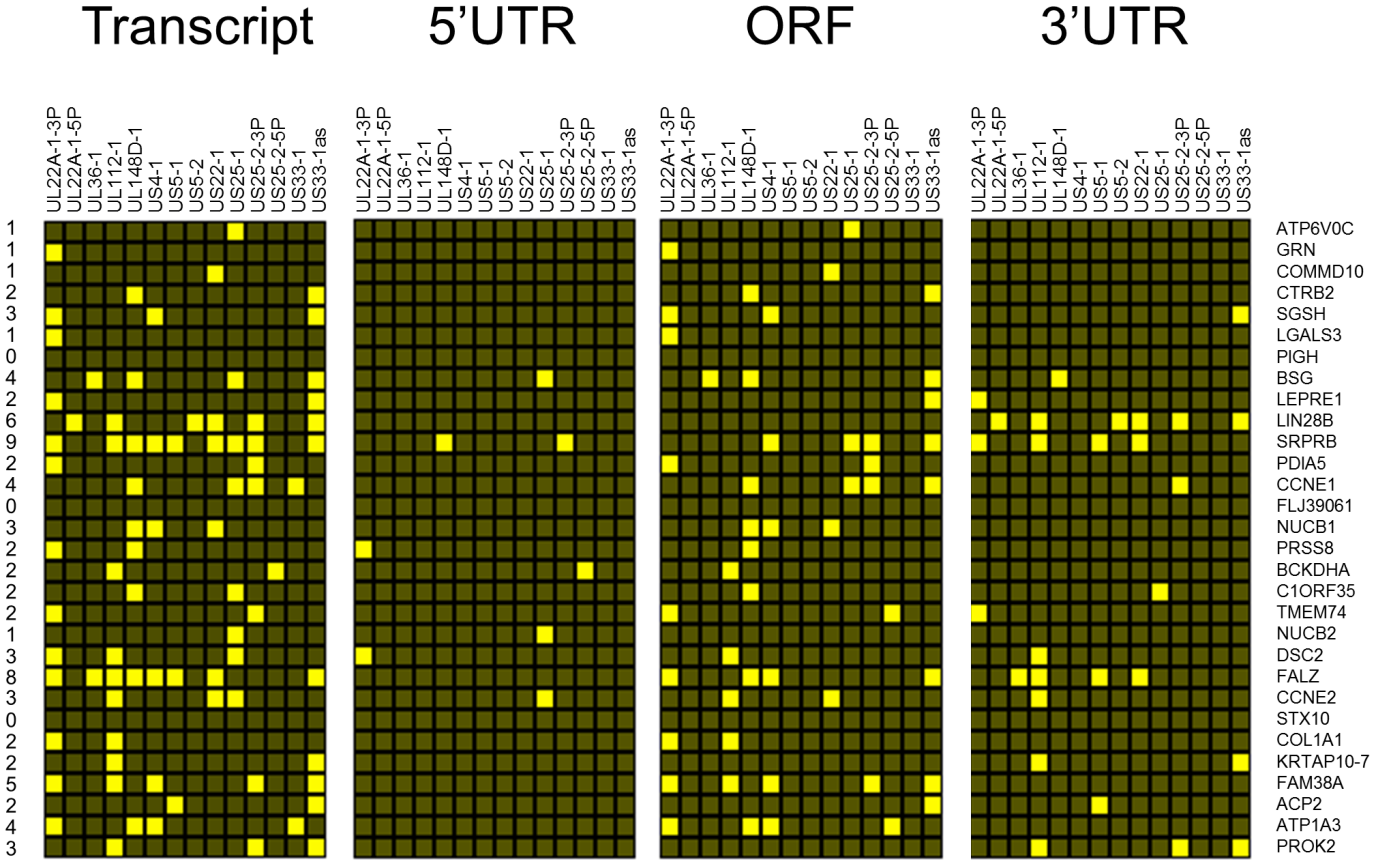
Top 30 enriched genes contain multiple target sites for HCMV miRNAs. Sequences for the top 30 enriched transcripts were analysed for HCMV miRNA targets using RNA Hybrid algorithm. Figure shows hit matrix where a yellow square indicates at least one target site for the indicated HCMV miRNA. Independent hit matrices shown for targets either within the whole transcript, or within the CDS, 5′UTR and 3′UTR. Total number of potential miRNAs targeting a transcript shown in the far left column.

As pull down experiments were performed in the context of viral infection, any one, or a combination of, HCMV encoded miRNAs could target the identified transcripts. It is also possible that transcripts could be enriched through targeting by an induced cellular miRNA. Target interaction between miRNAs and transcripts rely heavily on binding between the 5′ end of miRNAs, specifically nucleotides 1 through 8, known as the seed sequence. To define which HCMV miRNA has the potential to target the identified transcripts we predicted seed match sites using the online algorithm RNAHybrid for the 14 most abundant HCMV miRNAs. Stringent parameters of full Watson Crick base pairing with bases 1–7 or 2–8 were employed with the top 30 putative targets analysed. All but three of the transcripts contained at least one seed match to the major HCMV encoded miRNAs, with most transcripts containing targets sites for multiple HCMV miRNAs (Figure 2). The majority of target sites reside within the open reading frame of the transcripts with only 14 of the 30 transcripts containing predicted seed matches for HCMV miRNAs within the 3′UTR. Although there is evidence that cellular miRNA targeting is heavily constrained to the 3′UTR region of transcripts [31]–[33], a number of studies, including our own, suggest that these constraints may not always be applied to viral miRNA targeting. In fact targeting by US25-1 was shown to predominantly target sites within the 5′UTR [29]. Full analysis of transcripts is shown in Supplemental Table S3.

**Figure 3:**
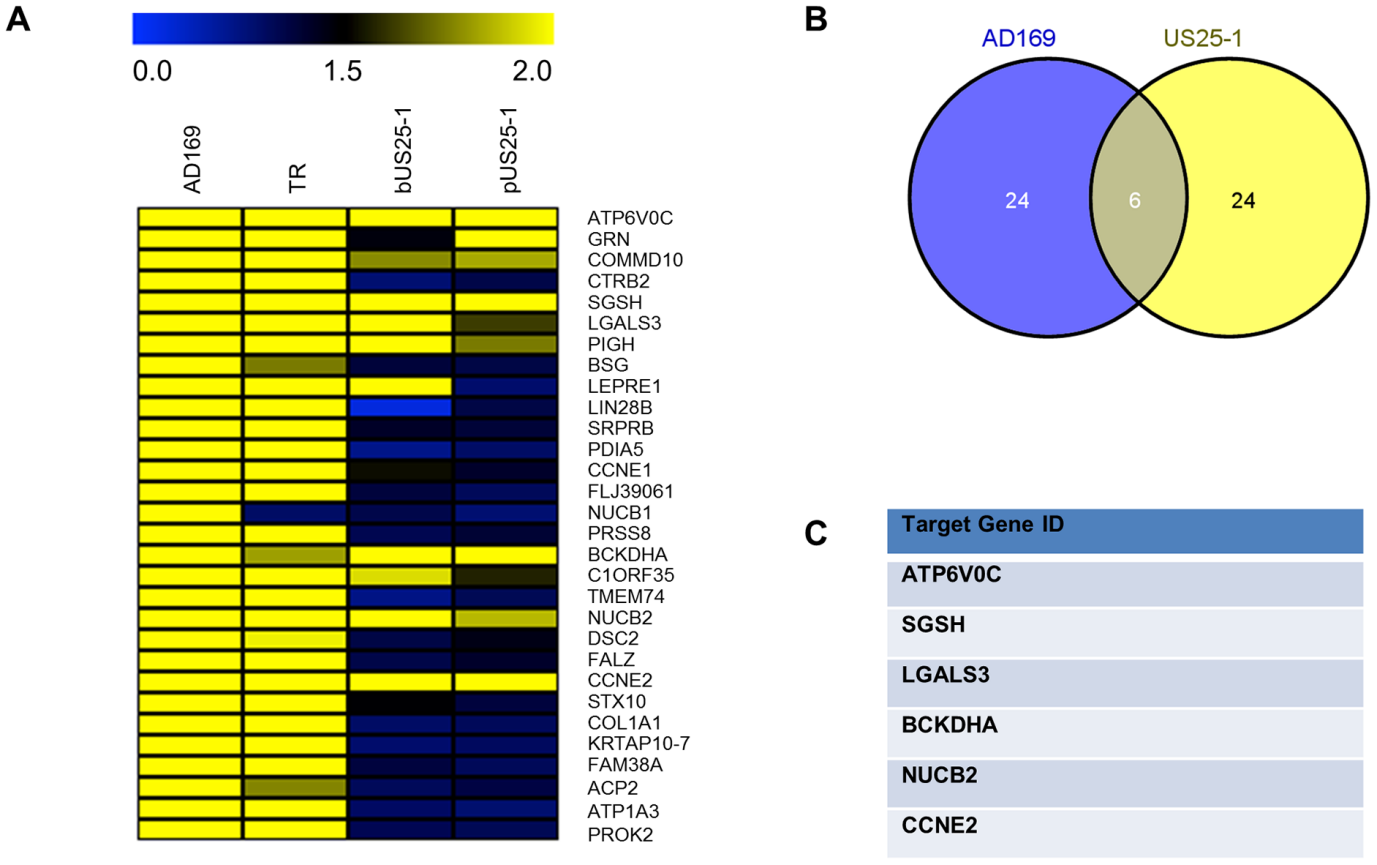
Comparison of RISC-IP profiles from infected fibroblast cells and HEK293 cells transfected with US25-1. (A) Heat map comparing the enrichment levels from fibroblast cells infected with HCMV or HEK293 cells transfected with either a plasmid expressing US25-1 (pUS25-1) or a US25-1 mimic (bUS25-1). (B) Venn diagram showing the overlap between the top 30 enriched genes from AD169 infected cells and combined enrichment data from cells transfected with US25-1. List of overlapping genes shown in (C).

To delineate the miRNA target interactions, we compared the RISC-IP data from infected cells with the previous published study generated from cells transfected with US25-1 [29]. Figure 3A represents heat map analysis comparing enrichment profiles from the top 30 enriched transcripts of cells infected with AD169 or TR, with cells transfected with either a plasmid expressing US25-1 or immunoprecipitations using a synthetic US25-1 mimic containing a biotin moiety. The majority of transcripts show clear enrichment with AD169 and TR as would be expected. In addition, highly enriched genes from infected cells were also significantly enriched in cells only expressing US25-1, demonstrating that these transcripts are targeted by US25-1 in the context of viral infection. Six genes, including the top target from the previous study, CCNE2, and the top target from this study, ATP6V0C, were in the top 30 enriched genes from cells infected with AD169 or cells transfected with US25-1 (Figure 3B and C).

**Figure 4:**
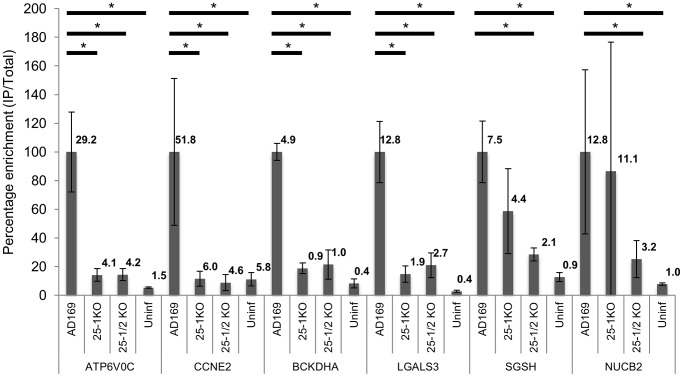
Deletion of US25-1 from HCMV results in loss of enrichment of identified targets. Enrichment of the six US25-1 targets was determined by RT-PCR following RISC-IP from cells infected with wild type virus or cells infected with US25 knock out viruses. Enrichment levels converted to percentage with enrichment from AD169 infected cells set at 100%. Raw enrichment values shown above each bar. Expression levels were corrected against GAPDH and each bar represents 3 biological repeats. * = statistically significant difference (p = <0.01). Statistics were performed on raw data using the Mann-Whitney non-parametric U-test.

Although the combined data sets provide strong evidence that these six genes are targeted by US25-1 in the context of virus infection, it is possible that other viral miRNAs may also target these genes, potentially complicating further validation. To determine whether this was the case, additional RISC-IP analysis was carried out comparing wild type AD169 virus to two recombinant AD169 viruses in which either US25-1 had been deleted, or the entire US25 region, encoding both US25-1 and 2, was deleted. Enrichment levels for each of the six genes identified from the previous analysis was determined by direct RT-PCR using specific primer probe sets (Figure 4). To allow direct comparison, enrichment values for wild type pull downs were set at 100% (actual enrichment values are displayed above each bar for reference). All six targets showed significant enrichment in infected cells compared to uninfected cells, validating the results from the original microarray experiments. Four of the six genes, ATP6V0C, CCNE2, BCKDHA and LGALS3, also showed a near complete loss of enrichment from cells infected with either US25 knock out viruses, indicating that US25-1 is required for enrichment of these transcripts. The results were less clear-cut for NUCB2 and SGSH. Although the levels of enrichment were reduced, the reduction was not statistically significant, suggesting that other viral miRNAs may be involved in targeting these genes. Additional validation of genes from the top 30 most enriched transcripts also showed significant enrichment in infected samples compared to uninfected samples, again validating the original microarray data (Supplemental Figure S2). However, no other genes showed a complete loss of enrichment in the knock out viruses. Transcripts that were not predicted to be targeted by US25-1, such as LIN28B, showed enrichment in both wild type and knock out virus, confirming that successful enrichment from the knock out virus infected cells had occurred. In addition no enrichment was detected in control transcripts such as beta actin (data not shown). No significant enrichment was observed following transfection with mimics corresponding to US25-2-3p, US25-2-5p or a US25-1 mimic containing a mutated seed region, demonstrating that neighboring miRNAs do not play a role in the enrichment of the six identified targets and that the seed region of US25-1 is necessary for effective enrichment (Supplemental Figure S3A).

**Figure 5:**
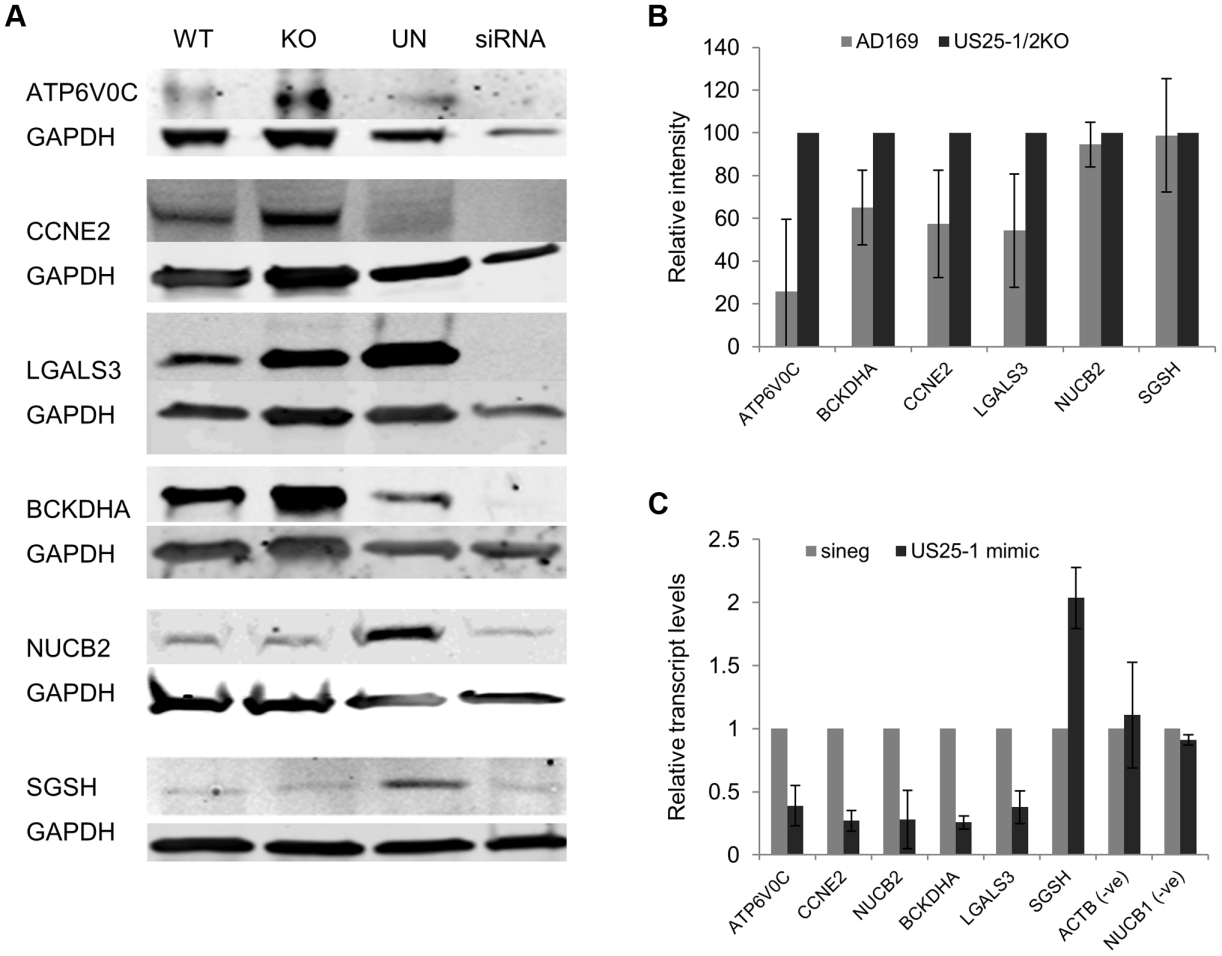
Deletion of US25-1 results in increased expression of identified targets in context of virus infection. (A) Fibroblast cells were infected at an MOI of 3 with either wild type virus or US25-1 knockout virus and harvested 72 hours post infection. Western blot analysis was performed using antibodies against identified US25-1 targets. Uninfected cells and cells transfected with siRNA against the gene being analysed are included for comparison. (B) Band intensities for three independent biological repeats were determined and corrected for GAPDH levels with relative intensities shown as a percentage with the uninfected value set to 100%. As data represents the ratio between wild type and infected protein levels the error of the ratio is incorporated into the error bars shown for the KO virus protein levels. (C) Fibroblast cells were transfected with either US25-1 mimic or a negative control mimic (40 nM). Cells were harvested 48 h post infection and RNA levels for each of the indicated transcripts determined by real time RT-PCR analysis. Relative levels of RNA for US25-1 mimic transfected cells compared to negative control transfected cells are shown with results normalized to GAPDH. Results represent 3 biological repeats.

Although these results confirmed that US25-1 RISC complexes bind to the identified transcripts it remained necessary to determine whether these interactions were functional and resulted in effects on gene expression. In our previous study we demonstrated that targeting of CCNE2 by US25-1 resulted in reduced cyclin E2 expression and conversely deletion of US25-1 from the virus resulted in increased expression of cyclin E2 in the context of virus infection. Using the same approach the effect of US25-1 on the expression levels of all six identified genes was investigated. Primary human fibroblast cells were infected at high MOI with either wild type AD169 or US25-1 KO virus and protein levels for the six genes compared by western blot analysis (Figure 5A). Uninfected cell lysates were also included as well as cells transfected with a siRNA specific for the target gene, to confirm the specificity of the antibody. As has been shown before, CCNE2 levels were higher in the knock out virus infected cells compared to the wild type infected cells. In addition, ATP6V0C, BCKDHA, LGALS3 all showed increased levels of expression in cells infected with the US25-1 knock out virus, whereas NUCB2 and SGSH did not show significant difference between wild type infected cells and knockout infected cells. Representative western blots are shown in Figure 5A with direct quantitation from three biological repeats shown in Figure 5B. These results correspond well with the RISC-IP data, indicating that ATP6V0C, CCNE2, BCKDHA and LGALS3 are targeted by US25-1 and deletion of this miRNA results in near complete abrogation of the inhibitory effects, whereas NUCB2 and SGSH may be targeted by additional viral miRNAs, or other virally induced mechanisms. Further supporting the role of US25-1 in targeting of these cellular genes, transfection of fibroblast cells with US25-1 mimic RNA results in significant knockdown at RNA levels of the putative US25-1 targets. The exception to this is SGSH, where transfection of US25-1 reproducibly resulted in a two-fold increase in total RNA levels. Whether this is due to a direct effect of US25-1 binding to the SGSH transcript or secondary effects from regulation of other US25-1 targets is unclear and will require further study. In addition, transfection of US25-2-3p and US25-2-5p did not result in significant knockdown of ATP6V0C, again supporting the role of US25-1 alone in targeting these genes (Supplemental Figure S3B).

**Figure 6:**
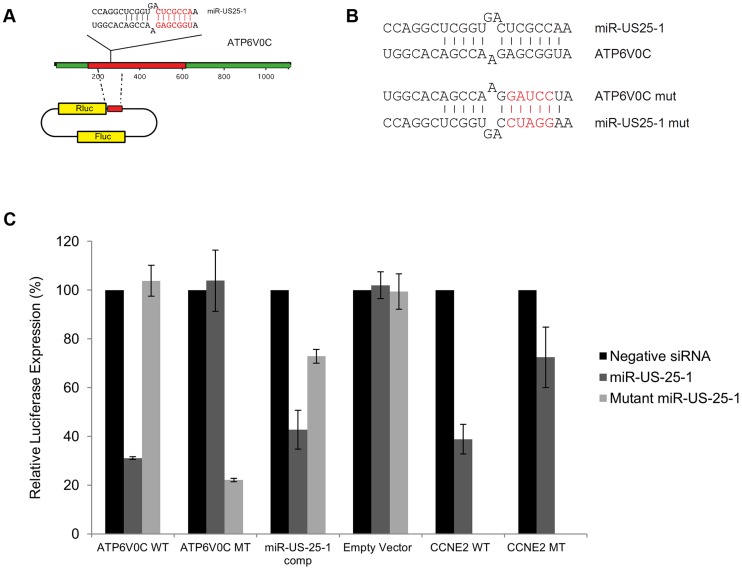
US25-1 targeting of ATP6V0C occurs through the predicted target site within the ORF. (A) The predicted US25-1 target site from ATP6V0C was cloned downstream of the luciferase reporter construct psiCheck2. The schematic shows the cloning strategy for ATP6V0C. (B) Schematic representation of sequence changes within the target site of ATP6V0C and the corresponding mutation in US25-1 mimic (sequence alterations in the US25-1 mimic are shown in red). (C) Constructs were co-transfected into HEK293 cells with US25-1 mimic, negative control siRNA or a US25-1 mimic with a mutated seed sequence at 40 nM. Data represents 3 biological replicates with standard deviation.

Our previous study showed that US25-1 predominantly targets the 5′UTR of transcripts and five of the six genes (including the previously identified CCNE2) have potential target sites within the 5′UTR for US25-1 (Supplemental Figure S5). However, ATP6V0C contains a 7mer target site for US25-1 downstream of the 5′UTR within the open reading frame. To determine whether the US25-1 target within the open reading frame is responsible for the observed knockdown in ATP6V0C protein expression we carried out luciferase assays using a construct containing the target site from ATPV0C cloned into the 3′UTR of the reporter construct psiCheck2 (Figure 6A). The construct was co-transfected into HEK293 cells with either US25-1 mimic, or a non-targeting control siRNA. A CCNE2 luciferase construct was included as a positive control. US25-1 mimic induced a significant reduction in luciferase expression, compared to the negative control siRNA for both constructs (Figure 6C). Furthermore, mutation of the ATP6V0C target seed region to a BamHI restriction site resulted in restoration of the luciferase activity, indicating that the target site identified in ATP6V0C is both sufficient and necessary for US25-1 specific inhibition of gene expression. Transfection of a US25-1 mimic with a mutated seed sequence that corresponds to the mutated ATP6V0C luciferase construct did not reduce expression of the wild type ATP6V0C luciferase construct, but did inhibit expression of the mutated ATP6V0C construct (Figure 6B and C).

**Figure 7:**
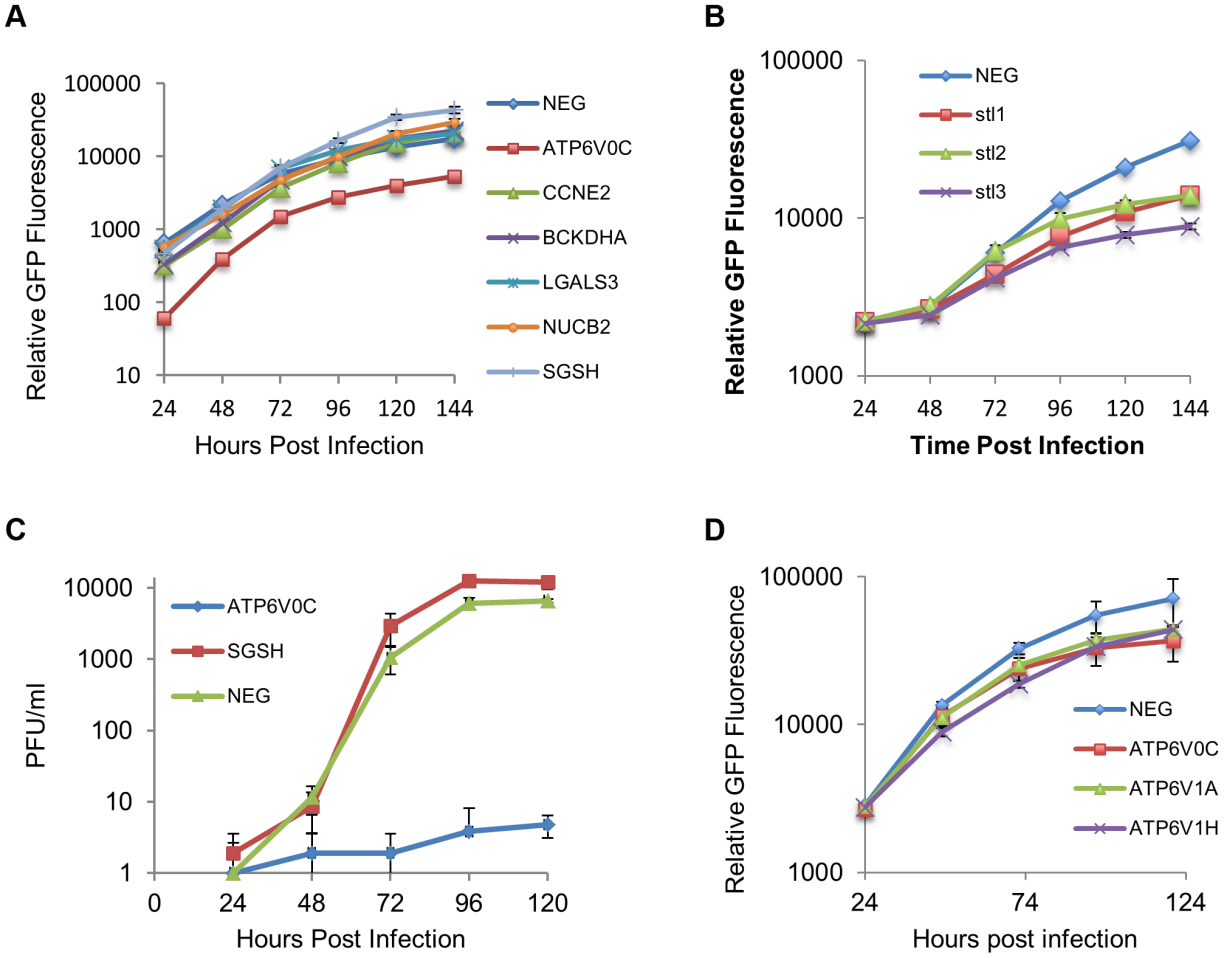
ATP6V0C is an essential host factor for HCMV replication. (A) Human Fibroblast cells were transfected with siRNAs (40 nM) against each of the six HCMV miRNA targets previously identified. Cells were then infected with a GFP tagged HCMV virus at an MOI of one, 16 hours post transfection. GFP levels were monitored during the course of infection and compared to determine the effect of knock down of the miRNA targets on HCMV replication. Data represents 4 biological repeats. (B) Fibroblast cells were transfected with three independent siRNAs targeting ATP6V0C (stl1, 2 and 3) as above. Each siRNA resulted in reduced virus growth as determined by GFP fluorescence. Data represents 4 biological repeats. (C) Cells were transfected with pooled siRNAs and infected as above with cells and supernatant harvested at the indicated time points and analysed for infectious virus by plaque assay. Data represents 3 biological repeats (D) Fibroblast cells were transfected with siRNAs as above targeting ATP6V1A or ATP6V1H, components of the V-ATPase complex and HCMV replication analysed by GFP fluorescence. Replication was inhibited by similar levels as observed for knockdown of ATP6V0C. Data represents 4 biological repeats.

Although it is clear that US25-1 targets and regulates the identified genes in the context of viral infection it is less clear whether these targets are functionally relevant. It is possible that viral miRNAs target many genes, but only a few are important to the virus while other targets represent fortuitous or irrelevant targets in the context of infection. Previous studies have established that cell cycle control and expression of cyclin E proteins are intimately involved in HCMV biology. However, the potential role of the remaining 5 targets is unclear, as they have not been reported as host factors involved in HCMV replication. To investigate their potential role, replication of HCMV was analysed following siRNA knockdown of each of the individual US25-1 targets in primary human fibroblast cells. Knock down of each gene was confirmed by RT-PCR (Supplemental figure S4A). Cells were infected, post siRNA transfection, at an MOI of 1 with the clinical strain TB40E, which expresses GFP fluorescence protein under control of the SV40 promoter. This allows continuous monitoring of virus levels through GFP fluorescence. As can be seen from Figure 7A, knockdown of SGSH resulted in a modest increase in virus replication. In contrast knockdown of ATP6V0C resulted in significant reduction in virus replication at all time points. To rule out the possibility that the effects on virus replication were caused by artifactual or non-targeting effects, the assay was repeated using three additional independent siRNAs targeting different regions of the ATP6V0C transcript (Figure 7B). All three siRNAs resulted in the same reduction of GFP fluorescence. To determine the effect on production of infectious virus, plaque assays were conducted following transfection of fibroblast cells with siRNA pools targeting ATP6V0C, SGSH or a negative control siRNA. The results support and confirm the GFP screen with a modest but statistically significant increase in replication in cells transfected with SGSH siRNA (Mann-Whitney U Test: p = 0.0039) and a more dramatic reduction in virus production in cells transfected with ATP6V0C siRNA (Figure 7C). In fact, knock down of ATP6V0C resulted in almost complete block in virus production, indicating that expression of ATP6V0C is essential for HCMV virus production and suggests that acidification of endosomal compartments is required for HCMV acute replication. Cell viability assays demonstrate that the reduction in virus replication was not due to cellular toxicity caused by ATP6V0C knockdown and transfection of the small RNAs did not induce an interferon response (Supplemental Figure S4B and C). However, previous reports have indicated that ATP6V0C may have functions independent of endosomal acidification [34]. To determine whether the observed inhibition of virus replication is due to a defect in endosomal acidification, fibroblast cells were transfected with siRNAs targeting ATP6V1A and ATP6V1H, components of the same vacuolar ATPase complex. Disruption of any of the essential components has been shown to be sufficient to destabilize the complex. Knockdown of either ATP6V1A or ATP6V1H resulted in a similar reduction in HCMV replication compared to cells in which ATP6V0C had been knocked down (Figure 7D). These results support the conclusion that acidification of the endosomal compartments by V- ATPase is essential for efficient HCMV replication and this gene is targeted by the HCMV miRNA US25-1.

## Discussion

Despite recent advances in our understanding of miRNA transcript interaction, identification of valid targets remains challenging. The nature of miRNA targeting, where functional effects may rely on multiple miRNAs targeting a single transcript or multiple genes within single pathways being targeted, requires a system wide approach to elucidate the functions of miRNAs. Recent studies have used such approaches to identify targets of gamma-herpesvirus miRNAs [30], [35]–[38]. However, no systematic screening approach has been presented for HCMV in the context of viral infection. Here we use a RISC-IP approach to identify putative targets of HCMV miRNAs in the context of viral infection, an important step towards generating a global understanding of the role these small regulatory RNAs play in the biology of HCMV and herpes viruses in general.

Using a laboratory strain of HCMV and clinical strain we identified a total of 906 transcripts that were enriched by at least two fold over immunoprecipitations from uninfected cells, 222 of which were enriched by both viruses. Relatively few cellular targets of HCMV miRNAs have been previously published [27], [39], [40]. Of those, BclAF1 and RANTES did not show significant enrichment in infected cells. In the case of RANTES and BclAF1 it is possible that the effects are cell type specific or the complex formed between the transcript and RISC is not stable and therefore does not result in enrichment. MICB was significantly enriched in both uninfected and infected cells correlating well with previous studies indicating that both cellular and viral miRNAs target this gene.

Many of the targets identified in this study have not previously been linked to HCMV, and in many cases, have not been linked to virus infections in general. Only a fraction of host genes have been investigated for potential roles in viral infections. Systematic analysis of viral miRNA targets can effectively exploit target identification for the discovery of novel host factors that play important roles in the biology of HCMV. Here we verify the effectiveness of this approach with the identification of at least two genes that have significant effects on HCMV replication. Knockdown of ATP6V0C resulted in attenuation of viral replication, while knockdown of SGSH resulted in an increase in viral replication.

The most highly enriched target identified in this study, ATP6V0C, is a component of the Vacuolar ATPase, which is responsible for acidification of endosomal compartments [41]. Knockdown of this gene resulted in striking inhibition of virus replication with almost no infectious virus detected during growth curve analysis. Acidification of endosomes has previously been shown to be required for HCMV entry into endothelial and epithelial cells through receptor mediated endocytosis. However, infection of fibroblast cells occurs through direct fusion with the plasma membrane and has been demonstrated to be pH independent [42]. The attenuation of HCMV replication through siRNA targeting of ATP6V0C is therefore unlikely to be due to a defect in viral entry. In support of this, although GFP levels were reduced in siRNA knockdown experiments, all cells were clearly GFP positive 24 hours post infection (Supplemental Figure S6). An alternative explanation could involve the marked reorganization of intracellular membranous organelles during the formation of HCMV assembly compartment [43]. A block in endosomal acidification may interfere with this process resulting in attenuation of virus replication and virion assembly. Interestingly, a previous report indicated that US25-1 expression has a negative effect on acute replication of HCMV [44]. This effect was not specific, as adenovirus replication was also inhibited, suggesting targeting of a cellular factor was responsible for the phenotypic effects. Our findings suggest this cellular factor may be ATP6V0C and acidification of endosomal compartments may be a necessary process for efficient replication of DNA viruses in general.

SGSH is involved in heparin sulphate degradation in the lysosomal compartment. Initial attachment of HCMV virions to target cells has been shown to occur through binding of viral glycoprotein B with heparin sulphate moieties on the cell surface [41]. However, it is unlikely that disruption of this pathway would result in higher levels of heparin sulphate on the cell surface. Western blot analysis in this study shows that infection with HCMV results in significant reduction in SGSH levels, and although this reduction appears to occur independently of US25-1, the result suggests targeting of this gene plays an important role in the replication of the virus.

The question remains as to why the virus would target a cellular gene, such as ATP6V0C, required for efficient replication. We previously demonstrated that UL112-1 attenuates HCMV replication through direct targeting of the immediate early gene IE72 and suggested that this represents a mechanism of establishing or maintaining viral latency [22]. Targeting of ATP6V0C may represent a similar mechanism, possibly blocking assembly and release of virions during latent infection. Alternatively, targeting by US25-1 may be unrelated to viral replication, but rather serve a different function such as immune evasion. Acidification has been shown to be required for efficient signaling by endosomal resident toll like receptors and for efficient MHC class II presentation [45], [46]. Blocking acidification of endosomes through targeting of ATP6V0C may be an effective way for the virus to interfere with both innate and adaptive immune response.

In conclusion, this study greatly increases the number of putative and validated targets of HCMV miRNAs. The use of systematic miRNA target analysis with focused siRNA screening is an effective strategy for the identification of novel host virus interactions. Finally the V-ATPase complex is an essential host factor in HCMV replication and is targeted by the HCMV miRNA US25-1.

## Materials and Methods

### Cells and viruses

Normal human dermal fibroblast (NHDF) cells (Clonetics) were cultured in Dulbecco's modified Eagle's medium supplemented with 10% fetal calf serum and penicillin-streptomycin-L-glutamine. HCMV strain AD169 was obtained from the American Type Culture Collection (Rockville, Md.). TR HCMV was obtained from Dr Jay Nelson. TB40E GFP was obtained from Dr Goodrum [47]. All HCMV strains were grown on primary fibroblast cells following infection at low MOI. Virus preps were purified over 10% sorbitol gradients.

### RISC-IP analysis

RISC-IP analysis was carried as out previously described [28], [48]. In brief for systematic analysis of HCMV miRNA targets, primary human fibroblast cells were infected at a MOI of three with either AD169 or TR. Three days post infection cells were lysed, samples taken for total RNA and miRNP complexes immunoprecipitated using anti Ago2 antibody followed by streptavidin bead pull down. RNA was isolated using Trizol and analyzed for quality using an Agilent Bioanalyzer and transcript levels determined on the Illumina HumanRef-8 platform. Microarray data was analyzed using Gene sifter software. Enrichment of specific transcripts, through association with miRNP complexes was determined by dividing the immunoprecipitated levels of transcripts by the total levels. Analysis of specific genes by RT-PCR was conducted using the same protocol and parameters, except specific primer probe sets were used instead of microarray analysis. Primer probe sets were purchased from Lifetechnologies. For mimic RISC-IPs the same procedure was followed except 293T cells were transfected with 40 nM of mimic RNA and cells were harvested 48 hours post transfection.

Argonaute specific antibody was generated by immunization of rabbits with a peptide corresponding to the N terminal region of Argonaute 2 (5-MYSGAGPALAPPAPPPPIQGYAFKPPPRPD3′).

### Sequence analysis

Transcript sequences were down loaded from NCBI using RefSeq ID's. Predicted binding between HCMV miRNAs and putative target transcripts were determined using the online algorithm RNAhybrid (http://bibiserv.techfak.uni-bielefeld.de/rnahybrid/) [49]. Parameters were selected to include Watson-Crick base pairing between either nucleotides 1 to 7 or 2 to 8. Full transcript data was searched for seed sequence matches using a Java based script program.

### miR-US25-1 and miR-US25-1/2 KO viruses

miR-US25-1 pre-miRNA coding region was deleted from AD169 BAC clone using BAC technology as previously described [50]. Briefly a PCR amplified cassette containing FRT flanked Kanamycin was recombined into AD169 BAC genome replacing the miR-US25-1 coding region using primers listed in Supplemental table S4.

Sequence in italics indicates regions homologous to FRT flanked Kanamycin cassette with remaining sequence homologous to recombination site in HCMV genome. The Kanamycin cassette was then removed by recombining the FRT sites through inducible FLIP recombinase. The resulting BAC was isolated and electroporated into human primary fibroblast cells to produce infectious virus. Schematic representations of recombination strategies are shown in Supplemental Figure S7.

### Small RNA transfections

Cells were transfected with small RNAs using RNAiMAX lipofectamine reagent (Life technologies) according to manufacturer's guidelines with the following modifications. Fibroblast cells were double transfected with 20 pmol (40 nM) of small RNA per 24 well 8 hours apart. Cells were either infected or harvested 24 hours post transfection for siRNAs or 48 hours post transfection for mimics. Control cells were transfected with a non-targeting negative control siRNA (Qiagen – cat 1027310). The sequence and siRNA IDs are listed in Supplemental Table S5.

### RT-PCR analysis

Total RNA was harvested using Trizol with concentrations and RNA quality determined by nano-drop spectrophotometer analysis. 100 ng of total RNA was DNAse treated (Promega) then reverse transcribed using high capacity cDNA reverse transcription kit (ABI). Real time PCR was carried out using gene specific primer probe sets from ABI on a Rotor gene 3000 (Corbet Research). Relative expression levels were determined by delta delta Ct calculation with levels corrected to GAPDH levels.

### Western blot analysis

Human primary fibroblast cells were grown in either 10% serum supplemented DMEM before infection at a multiplicity of 3 with either wild type AD169, miR-US25-1, or miR-US25-1/2 knock out virus. 72 hours post infection, cells were harvested using SDS sample loading buffer. 30 ul of protein sample were loaded and proteins were probed using primary antibodies to ATP6V0C (Aviva), BCKDHA (Cambridge Biosciences), CCNE2 (Abcam), LGALS3 (Cambridge Biosciences), NUCB2 (Sigma), and SGSH (Genetex) according to manufacturer's specifications. Protein loading was normalised to GAPDH (Sigma). IR800 or IR680 dye conjugated anti-rabbit IgG and anti-mouse IgG secondary antibodies were purchased from LiCor. Blots were imaged using infrared fluorescence of appropriately tagged secondary antibodies and quantified using a LiCOR Odyssey scanner and software.

### Luciferase assay

ATP6V0C luciferase constructs were created using custom oligonucleotides corresponding to the genomic region between nucleotides 146 and 226 downstream of the transcriptional start site, flanking the bioinformatically predicted miR US25-1 target site (GAGCGGT starting at nucleotide 186). For the ATP6V0C mutant construct, the miR US25-1 target site was replaced with a BAMHI restriction site. These inserts were cloned downstream of the renilla luciferase reporter gene of the pSicheck 2 dual luciferase construct (Promega). Cloning oligonucleotides are shown in Supplemental table S4 Luciferase constructs were co-transfected with miR US25-1 mimic or control mimic (IDT) into HEK293 cells using Lipofectamine 2000 reagent according to the manufacturer's instructions. Cells were harvested 48 hours post transfection and luciferase levels measured using Promega's dual luciferase reporter kit. CCNE2 luciferase constructs were created and assays were performed as described previously [29].

### Virus growth curves

For virus growth curve analysis by GFP fluorescence 96 well plates seeded with primary human fibroblast cells were transfected with siRNAs at a final concentration of 2 nM using RNAiMAX transfection reagent (Life Technologies). Specific siRNAs for ATP6V0C (S80), ATP6V1A, ATP6V1H BCKDHA, CCNE2, LGALS3, NUCB2, and SGSH were obtained from Life Technologies. 24 hours post transfection, cells were infected at a MOI of 1. The MOI was empirically determined to provide robust signal without inducing extensive cell death through CPE. Twenty-four hours post infection cells were washed three times and overlayed with fresh complete DMEM media without phenol red pH indicator (Lonza) and GFP levels monitored using Biotech Synergy HT plate reader. For plaque assays 24 well plates seeded with HCMV were transfected with pooled siRNAs for ATP6V0C (Life Technologies) and SGSH (Thermo Scientific). 24 hours post transfection, cells were infected at an MOI of 1. 24 hours post infection cells were washed three times and at indicated time points the cell monolayer was scraped into the media and the media and cells collected and frozen. Standard plaque assays were carried out on human primary fibroblast cells overlayed with carboxy methyl cellulose.

## Supporting Information

Figure S1 **Example calculation of corrected enrichment of miRNA target transcripts.** PSG6 gives a false positive because, although IP levels remain the same for both Uninfected and infected, total levels are reduced drastically in infected sample resulting in false enrichment. As total levels are also reduced in the Pre-Serum sample this counters the false enrichment through the correction calculation.
(TIF)

Figure S2 **Validation of RISC-IP enrichment by RT-PCR.** Enrichment of selected top 30 targets was determined by RT-PCR following RISC-IP from cells infected with wild type virus or cells infected with US25 knock out viruses. Enrichment levels converted to percentage with enrichment from AD169 infected cells set at 100%. Raw enrichment values shown above each bar. Expression levels were corrected against GAPDH and each bar represents 3 biological repeats. * = statistically significant difference (p = <0.01). Statistics were performed on raw data using the Mann-Whitney non-parametric U-test.
(TIF)

Figure S3 **US25-2-3p and US25-2-5p do not target ATP6V0C.** (A) Enrichment of selected top 30 targets was determined by RT-PCR following RISC-IP from cells transfected with indicted miRNA mimics (40 nM), including US25-1 mutant seed mimic. Enrichment levels converted to percentage with enrichment from AD169 infected cells set at 100%. Raw enrichment values shown above each bar. Expression levels were corrected against GAPDH and each bar represents 3 biological repeats. * = statistically significant difference (p = <0.01). Statistics were performed on raw data using the Mann-Whitney non-parametric U-test. (B) RNA levels were determined for ATP6V0C by RT-PCR, following transfection of fibroblast cells with US25-1, US25-2-3p or US25-2-5p. RNA levels were normalized to GAPDH and compared to cells transfected with a US25-1 seed mutant mimic.
(TIF)

Figure S4 **Confirmation of siRNA activity.** (A) Percentage knock down of each of the target transcripts is shown following transfection of human fibroblast cells with 40 nM of siRNA. Cells were harvested 24 hours post transfection and RT-PCR analysis performed as previously described. Percent knockdown versus cells transfected with negative control siRNA is shown. All assays were normalized against GAPDH levels and assays performed in triplicate. Stl1, 2 and 3 represent the independent siRNAs from the STL pool, targeting ATP6V0C (B) Human fibroblast cells were transfected with 40 nM siRNA or mimic and cells harvest 48 hours post transfection. Total RNA was harvested and IFN beta levels determined by RT-PCR. Positive control was RNA from cells transfected with non-infectious viral RNA. ND indicates not detected. (C) Cytotoxic effects on cells transfected with ATP6V0C siRNAs was measured using CytoTox-Glo according to manufacturer's instructions. Fibroblast cells were transfected at 40 nM and harvested 48 hours post transfection. Results are shown as relative percentage of luciferase.
(TIF)

Figure S5 **Schematic representation of US25-1 targets.** Untranslated regions of transcripts are shown in red, with translated region of transcript shown in green. Seed region of miRNA target interaction highlighted in red.
(TIF)

Figure S6 **Reduction of HCMV replication from ATP6V0C knock down is not due to block in viral entry.** GFP fluorescence is shown 24 hours post infection of primary fibroblast cells with GFP tagged HCMV. Cells were transfected with either negative control siRNA (A) or ATP6V0C siRNA (B) and infected 16 hours post transfection.
(TIF)

Figure S7 **Schematic diagram of knock virus construction.** Deletion of US25-1 or US25-1 and 2 sequence regions are shown as well as the recombination event removing the KAN cassette resulting in the final BAC constructs. Red boxes indicate the homologous regions of sequence where recombination occurs. Flanking transcripts US24 and US26 are shown in green.
(TIF)

Table S1 **Full data set for RISC-IP analysis of AD169 infected cells.** Signal levels for total RNA and IP RNA levels are shown from uninfected and infected pull down experiments. Final enrichment level represents analysed data after correction for false enrichment as explained in supplemental figure S1.
(ZIP)

Table S2 **Full data set for RISC-IP analysis of TR infected cells.** Data analyzed as for supplemental table S1.
(ZIP)

Table S3 **Full analysis of transcripts for HCMV miRNA seed targets.** Transcript sequences were down loaded from NCBI using RefSeq ID's. Transcript data sets were searched for seed sequence matches using a Java based script program. Seed matches for either 1 to 7 or 2 to 8 nucleotides are given. Results are shown for either the ORF, 5′ or 3′ UTR regions of the transcripts. The position of the target site is given as nucleotide coordinates.
(XLSX)

Table S4 **Summary of cloning oligonucleotides.** The US25-1 target seed region for ATP6V0C is highlighted in yellow and the sequence changes to create the mutant seed region are indicated in red.
(DOCX)

Table S5 **Small RNA sequences.** Sequences of siRNA and mimics are shown along with assay ID numbers. For SGSH, a Dharmacon smart pool was used and target sequence is shown under sense strand column. The seed mutation in US25-1 is indicated in red.
(DOCX)
