# Supplementary figures and images for: Systematic MicroRNA Analysis Identifies ATP6V0C as an Essential Host Factor for Human Cytomegalovirus Replication
Source: PLoS Pathog. 2013 Dec 26;9(12):e1003820. doi: 10.1371/journal.ppat.1003820 (PMC3873435; doi:10.1371/journal.ppat.1003820)

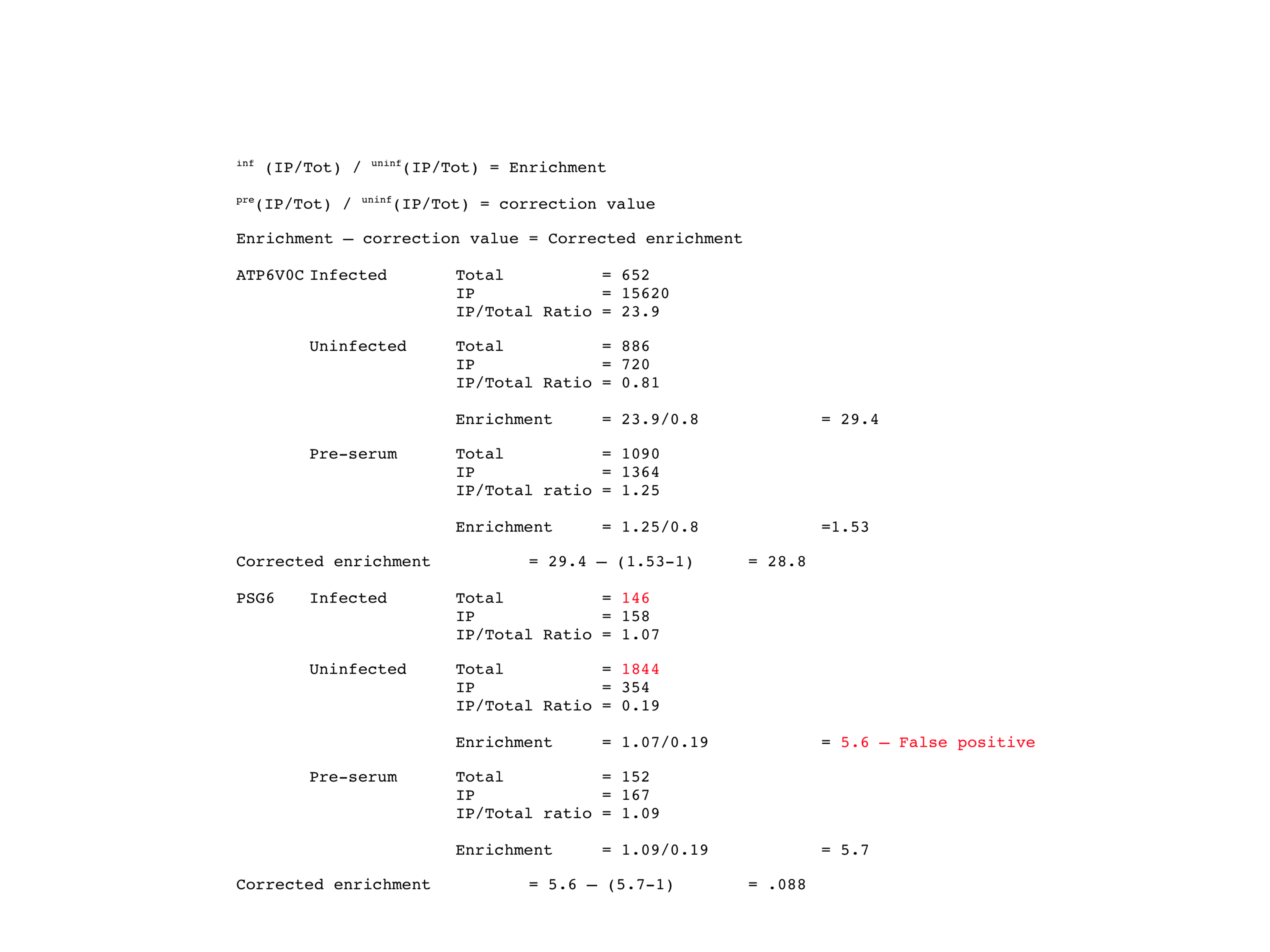

Supplement: Figure S1 — Example calculation of corrected enrichment of miRNA target transcripts. PSG6 gives a false positive because, although IP levels remain the same for both Uninfected and infected, total levels are reduced drastically in infected sample resulting in false enrichment. As total levels are also reduced in the Pre-Serum sample this counters the false enrichment through the correction calculation. (TIF) [file ppat.1003820.s001.tif]

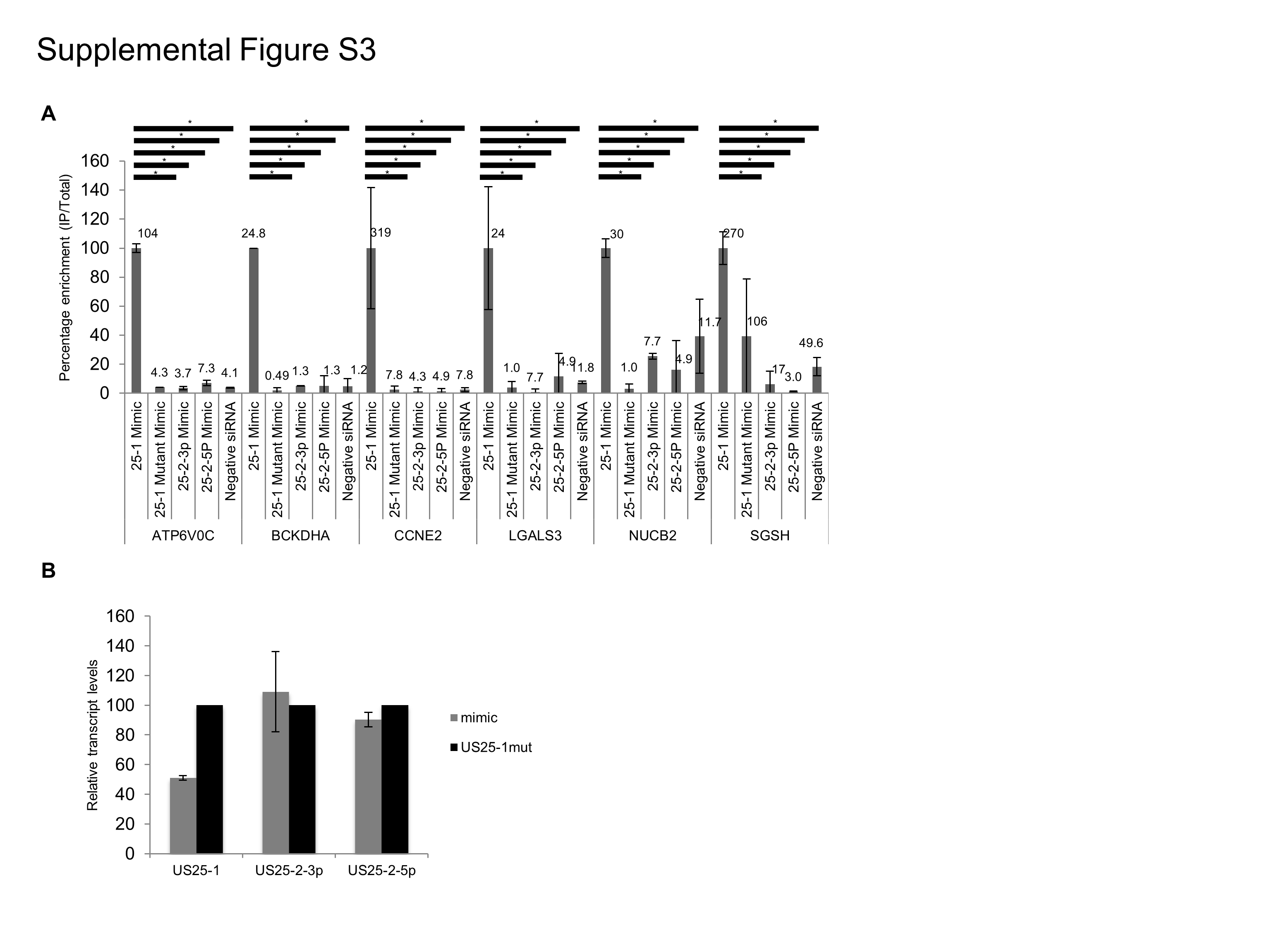

Supplement: Figure S3 — US25-2-3p and US25-2-5p do not target ATP6V0C. (A) Enrichment of selected top 30 targets was determined by RT-PCR following RISC-IP from cells transfected with indicted miRNA mimics (40 nM), including US25-1 mutant seed mimic. Enrichment levels converted to percentage with enrichment from AD169 infected cells set at 100%. Raw enrichment values shown above each bar. Expression levels were corrected against GAPDH and each bar represents 3 biological repeats. * = statistically significant difference (p = <0.01). Statistics were performed on raw data using the Mann-Whitney non-parametric U-test. (B) RNA levels were determined for ATP6V0C by RT-PCR, following transfection of fibroblast cells with US25-1, US25-2-3p or US25-2-5p. RNA levels were normalized to GAPDH and compared to cells transfected with a US25-1 seed mutant mimic. (TIF) [file ppat.1003820.s003.tif]

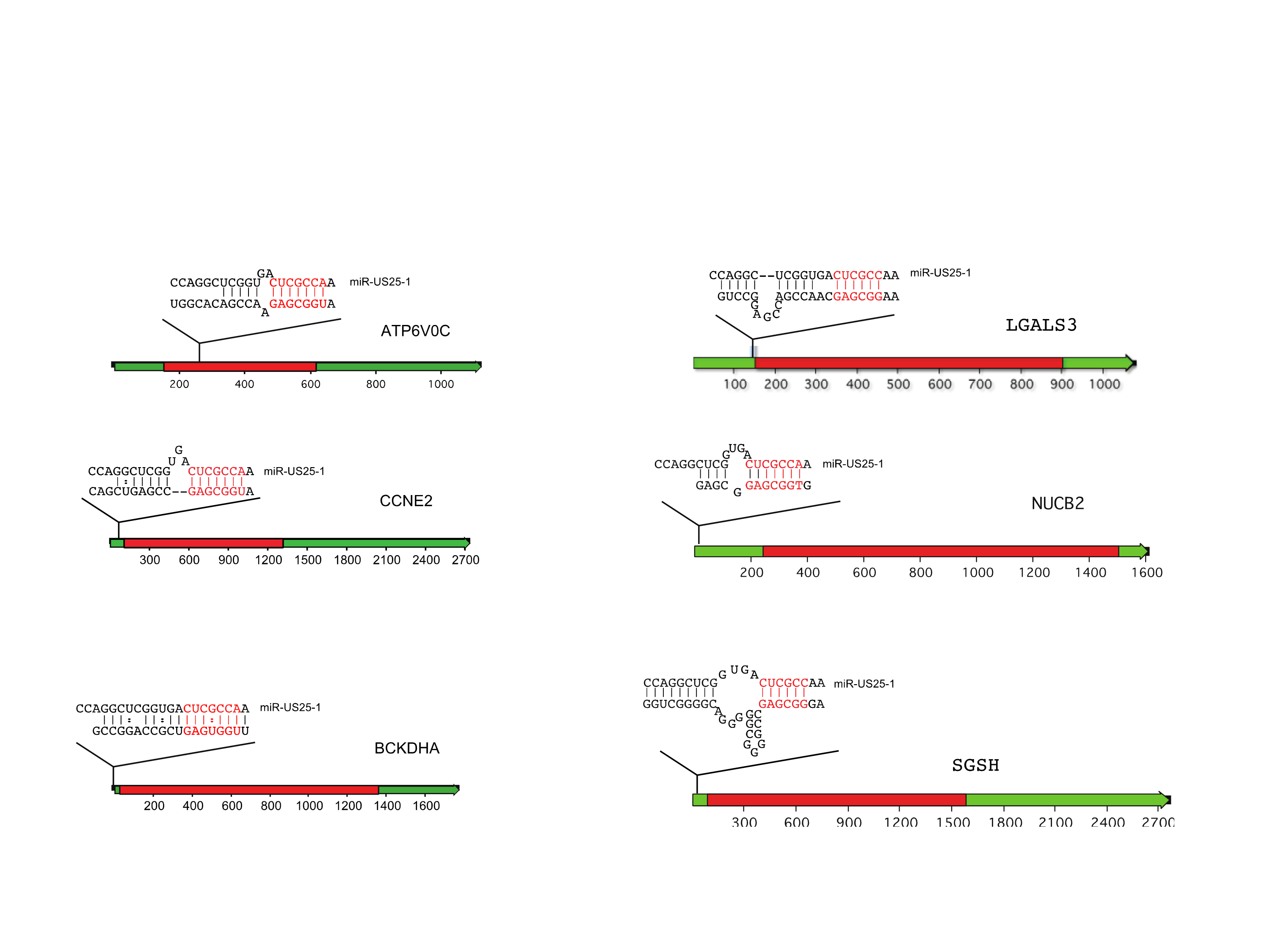

Supplement: Figure S5 — Schematic representation of US25-1 targets. Untranslated regions of transcripts are shown in red, with translated region of transcript shown in green. Seed region of miRNA target interaction highlighted in red. (TIF) [file ppat.1003820.s005.tif]

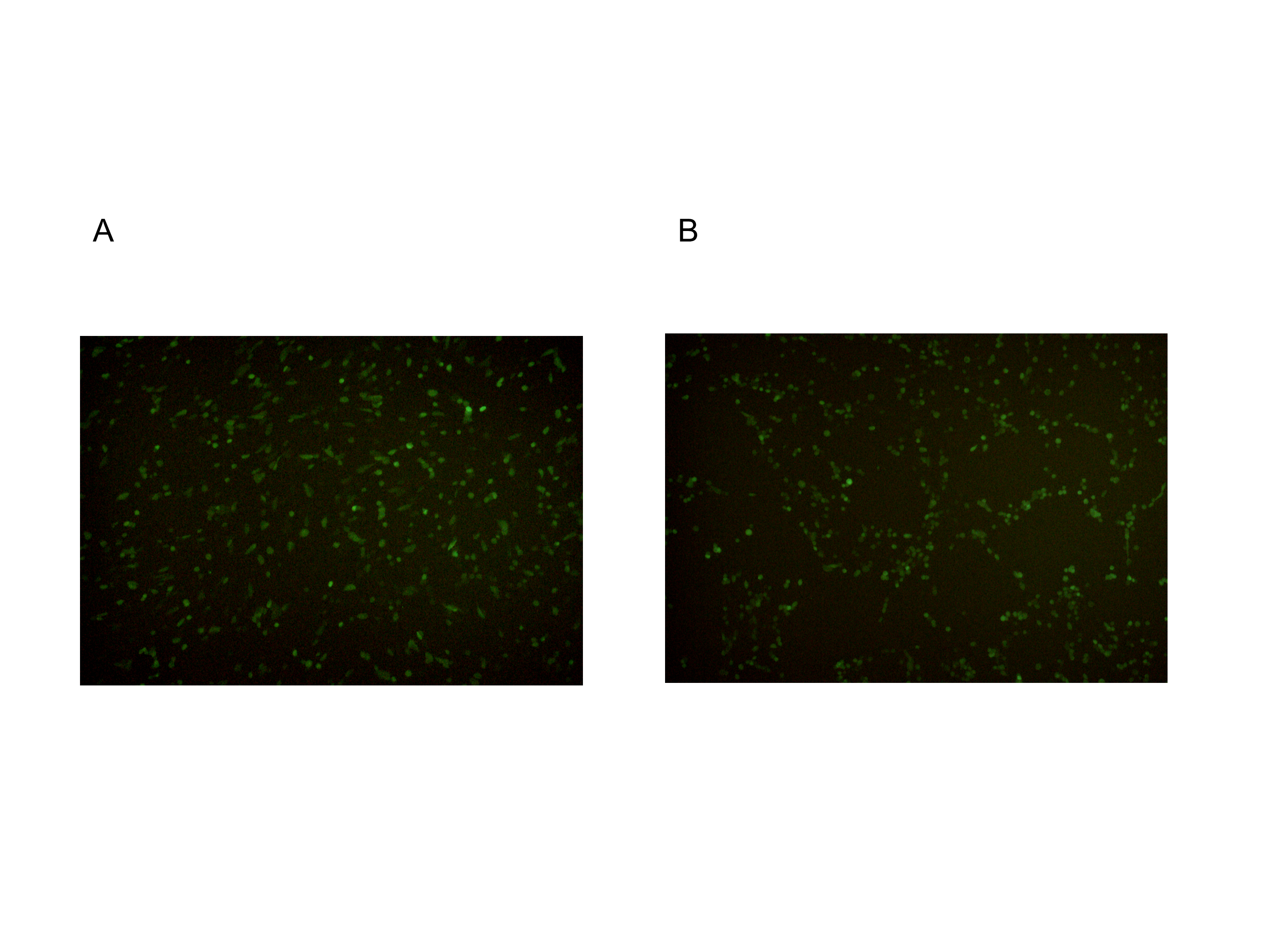

Supplement: Figure S6 — Reduction of HCMV replication from ATP6V0C knock down is not due to block in viral entry. GFP fluorescence is shown 24 hours post infection of primary fibroblast cells with GFP tagged HCMV. Cells were transfected with either negative control siRNA (A) or ATP6V0C siRNA (B) and infected 16 hours post transfection. (TIF) [file ppat.1003820.s006.tif]

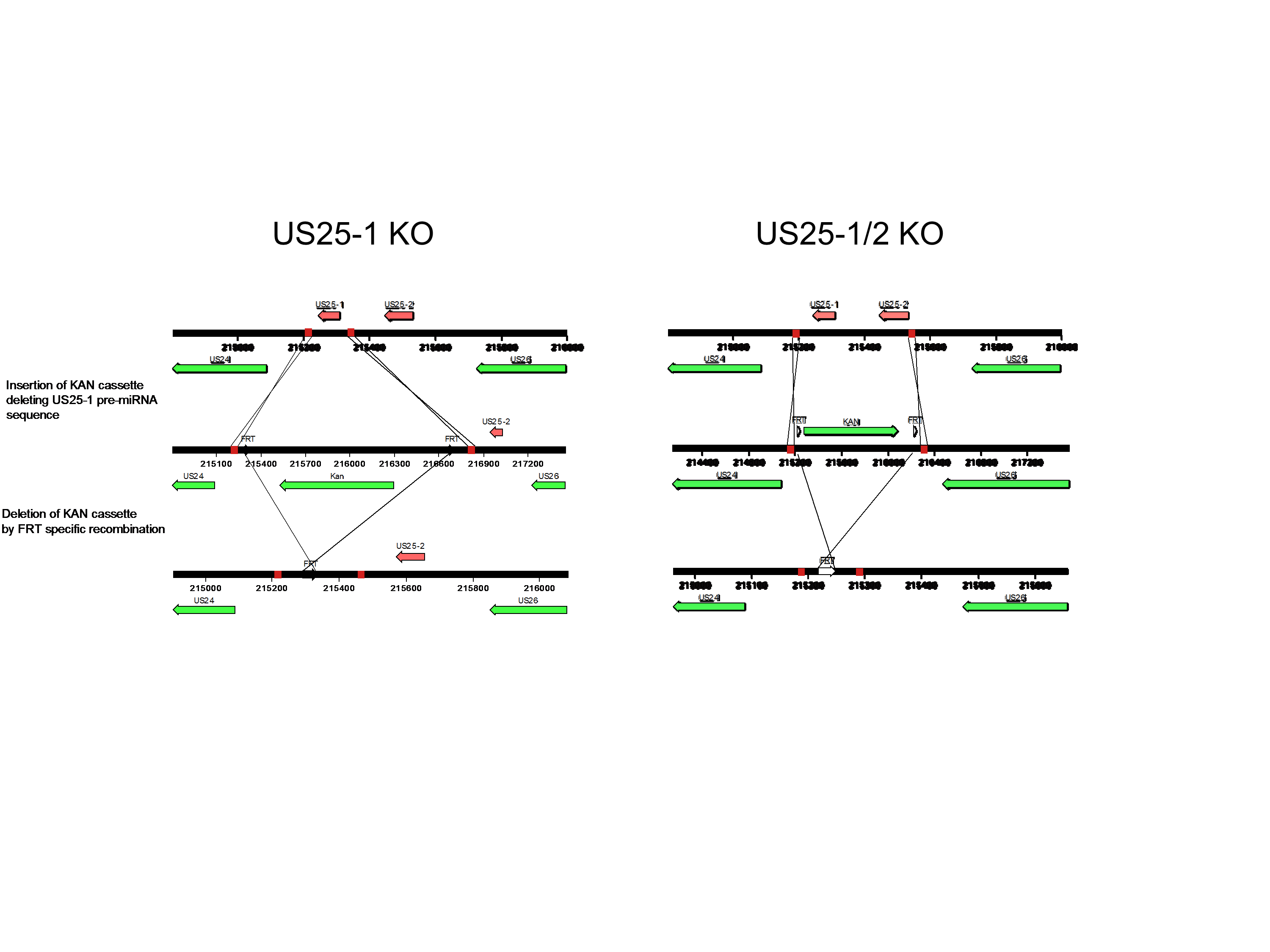

Supplement: Figure S7 — Schematic diagram of knock virus construction. Deletion of US25-1 or US25-1 and 2 sequence regions are shown as well as the recombination event removing the KAN cassette resulting in the final BAC constructs. Red boxes indicate the homologous regions of sequence where recombination occurs. Flanking transcripts US24 and US26 are shown in green. (TIF) [file ppat.1003820.s007.tif]
