# Supplementary material for: Systematic MicroRNA Analysis Identifies ATP6V0C as an Essential Host Factor for Human Cytomegalovirus Replication
Source: PLoS Pathog. 2013 Dec 26;9(12):e1003820. doi: 10.1371/journal.ppat.1003820 (PMC3873435; doi:10.1371/journal.ppat.1003820)
Supplement: Table S4 — Summary of cloning oligonucleotides. The US25-1 target seed region for ATP6V0C is highlighted in yellow and the sequence changes to create the mutant seed region are indicated in red. (DOCX) [file ppat.1003820.s011.docx]

| Oligo Name | Sequence |
| --- | --- |
| ATP6V0C target f | CTCGAGTCTTCAGCGCCCTGGGCGCTGCCTATGGCACAGCCAAGAGCGGTACCGGCATTGCGGCCATGTCTGTCATGCGGCCGGAGCGCGGCCGC |
| ATP6V0C target r | GCGGCCGCGCTCCGGCCGCATGACAGACATGGCCGCAATGCCGGTACCGCTCTTGGCTGTGCCATAGGCAGCGCCCAGGGCGCTGAAGACTCGAG |
| ATP6V0C mutant f | CTCGAGTCTTCAGCGCCCTGGGCGCTGCCTATGGCACAGCCAAGGATCCTACCGGCATTGCGGCCATGTCTGTCATGCGGCCGGAGCGCGGCCGC |
| ATP6V0C mutant r | GCGGCCGCGCTCCGGCCGCATGACAGACATGGCCGCAATGCCGGTAGGATCCTTGGCTGTGCCATAGGCAGCGCCCAGGGCGCTGAAGACTCGAG |
| US25-1 KO f | AAGCAACGCTACACCGTCACCCCGCTCCCAAGCGCCGCGGA*GTAAAACGACGGCCAGT* |
| US25-1 KO r | AAACTGGGGCGGGCGCGGGGTGGCGAAGCGGGAAGCGCT*CAGGAAACAGCTATGAC* |
| US25-1/2 KO r | GACGTCGGGACCGACGGACGCGACTCGGGGTCCTTCGGTT*CAGGAACACTTAACGGCTGA* |
