# Supplementary material for: Systematic MicroRNA Analysis Identifies ATP6V0C as an Essential Host Factor for Human Cytomegalovirus Replication
Source: PLoS Pathog. 2013 Dec 26;9(12):e1003820. doi: 10.1371/journal.ppat.1003820 (PMC3873435; doi:10.1371/journal.ppat.1003820)
Supplement: Table S5 — Small RNA sequences. Sequences of siRNA and mimics are shown along with assay ID numbers. For SGSH, a Dharmacon smart pool was used and target sequence is shown under sense strand column. The seed mutation in US25-1 is indicated in red. (DOCX) [file ppat.1003820.s012.docx]

| Small RNA | Assay ID | Sense | Anti Sense |
| --- | --- | --- | --- |
| Negative Control | 1027310 | UUCUCCGAACGUGUCACGUdTdT | ACGUGACACGUUCGGAGAAdTdT |
| ATP6V0C S80 | S80 | ACUGGAUGUUUAUUUAUAATT | UUAUAAAUAAACAUCCAGUAA |
| ATP6V0C stealth 1 | HSS141330(3 | GCCTATGGCACAGCCAAGAGCGGTA |  |
| ATP6V0C stealth 2 | HSS141332(3 | TCGTCGCCCTCATCCTCTCCACAAA |  |
| ATP6V0C stealth 3 | HSS182300 | CGGAGCAGATCATGAAGTCCATCAT |  |
| ATP6V1A | s1791 | GGUAAGGUAGAGUCAAUUATT | UAAUUGACUCUACCUUACCAT |
| ATP6V1H | s28403 | GGCUAUGAUUCAGUGCAAATT | UUUGCACUGAAUCAUAGCCAG |
| BCKDHA | s1909 | UGACACUGCUUAACACCAUTT | AUGGUGUUAAGCAGUGUCATG |
| CCNE2 | s17449 | CCAUUGAAGUGGUUAAGAATT | UUCUUAACCACUUCAAUGGAG |
| LGALS3 | s8148 | GGAGAGUCAUUGUUUGCAATT | UUGCAAACAAUGACUCUCCTG |
| NUCB2 | s9773 | GGAUUCCCUUCAAGAUAUATT | UAUAUCUUGAAGGGAAUCCAA |
| SGSH Stealth | HSS109691 | CCTTTCCCATCGACCAGGACTTCTA |  |
| SGSH | M-009053-01-0003 | CCGCAUGGACCAAGGAGUU |  |
|  |  | GAGAGUGGCGCGUACAACA |  |
|  |  | CGGAACAUCACUAGAAUUA |  |
|  |  | ACAAGGACCUCCGUCAUUA |  |
| US25-1 |  | AACCGCUCAGUGGCUCGGACCGC | GGUCCGAGCCACUGAGCGGGUCA |
| US25-1mut |  | AAGGAUCCAGUGGCUCGGACCGC | GGUCCGAGCCACUGGAUCCGUCA |
| US25-2-3p |  | GCGGGAGCUCUCCAAGUGGCUAG | AUCCACUUGGAGAGCUCCCGCGG |
| US25-2-5p |  | AGCGGUCUGUUCAGGUGGAUGA | AUCCACCUGAACAGACCGCU |
